# Morally excused but socially excluded: Denying agency through the defense of mental impairment

**DOI:** 10.1371/journal.pone.0272061

**Published:** 2022-07-26

**Authors:** Melissa de Vel-Palumbo, Rose Ferguson, Chelsea Schein, Melissa Xue-Ling Chang, Brock Bastian

**Affiliations:** 1 College of Business, Government and Law, Flinders University, Adelaide, South Australia, Australia; 2 School of Science, Psychology and Sport, Federation University, Melbourne, Victoria, Australia; 3 Department of Psychology and Neuroscience, University of North Carolina at Chapel Hill, Chapel Hill, North Carolina, United States of America; 4 School of Psychology, University of Queensland, Brisbane, Queensland, Australia; 5 School of Psychological Sciences, University of Melbourne, Melbourne, Victoria, Australia; University of Padova, ITALY

## Abstract

Defendants can deny they have agency, and thus responsibility, for a crime by using a defense of mental impairment. We argue that although this strategy may help defendants evade blame, it may carry longer-term social costs, as lay people’s perceptions of a person’s agency might determine some of the moral rights they grant them. Three randomized between-group experiments (*N* = 1601) used online vignettes to examine lay perceptions of a hypothetical defendant using a defense of mental impairment (versus a guilty plea). We find that using a defense of mental impairment significantly reduces responsibility, blame, and punitiveness relative to a guilty plea, and these judgments are mediated by perceptions of reduced moral agency. However, after serving their respective sentences, those using the defense are sometimes conferred fewer rights, as reduced agency corresponds to an increase in perceived dangerousness. Our findings were found to be robust across different types of mental impairment, offences/sentences, and using both manipulated and measured agency. The findings have implications for defendants claiming reduced agency through legal defenses, as well as for the broader study of moral rights and mind perception.

## Introduction

A defense of mental impairment—available in most Western democratic legal systems [1]—recognizes the principle that people lacking full mental capacity should be excused from criminal responsibility. But using this defense may come with a hidden cost. In denying their *agency* (the capacity to engage in intentional action [2]) mentally impaired defendants may initially absolve themselves of blame, but they may also lose certain rights and freedoms conferred to moral agents. The current research examines the potential negative consequences of lost agency for those using the defense of mental impairment, in terms of others’ moral regard of such defendants.

### Mental impairment excuses criminal responsibility through denial of agency

Defenses of mental impairment (defined differently across jurisdictions) typically involve the denial of one’s ability to reason and/or exercise full control over one’s actions at the time of the alleged crime, and thus constitute a denial of *mens rea* (a “guilty mind”)—a necessary requirement for establishing guilt [3, 4]. While major mental illnesses represent the classic case for a defense of mental impairment, brain injury and physical diseases affecting brain function may also constitute impairment. Across different jurisdictions, criteria for mental impairment defenses typically involve either a cognitive (not knowing the nature of one’s act) or volitional (not being able to control their behavior) element [3, 5, 6]. In the absence of these capacities, the law deems criminal responsibility and punishment to be inappropriate and unjustified.

In addition to a full defense, mental impairment is factored into judicial decision-making through a range of legal provisions that can reduce blame for a crime [7–9]. A partial defense (i.e., diminished capacity) due to mental impairment is available in many jurisdictions. Alternatively, a different plea, often termed “guilty but mentally ill” may impose a less stringent test of mental impairment, acknowledging that a defendant’s mental illness substantially contributed to his/her criminal conduct to some degree (for which he/she is nonetheless accountable). Last, mental impairment may be considered a mitigating factor in sentencing. All of these provisions seek to account for the reduced responsibility of mentally ill offenders even when they fall short of the criteria for a full defense of mental impairment; a full defense is the most complete abdication of agency and thus a useful starting point for exploration.

Consistent with the legal rationale for the defense of mental impairment, empirical research suggests that people attribute less blame and punishment to defendants with a mental illness [10–15] (but see [16, 17]). For example, Monahan and Hood [18] presented participants with case descriptions of a violent crime in which the offender either had or did not have a history of psychological disorder. Offenders with a history of psychological disorder were attributed with less free will and were considered less blameworthy and deserving of punishment. Similarly, Kleinke and Baldwin [19] demonstrated that persons supplying “crazy” explanations for criminal acts were ascribed less responsibility and intent, and were given shorter prison sentences than those providing “sane” explanations.

If mental illness reduces perceived blameworthiness, it is likely through reduced perceptions of *moral agency*—a prerequisite for moral responsibility. Agency requires first and foremost the ability to regulate one’s actions (e.g., through self-control, planning, and intention; this constitutes a volitional element [20]). In addition, agency also includes an understanding of what one is doing (e.g., perception, knowledge of norms, reason and thought; constituting a cognitive element typically referred to as moral knowledge [21]). Both correlational and experimental studies have shown that perceptions of agency (e.g., controllability of behavior) are related to perceptions of moral responsibility, deservingness of blame, and punishment for wrongdoing [20, 22–28].

Accordingly, insofar as mental impairment leads to perceived lack of agency, those invoking the defense should be perceived as less blameworthy for their criminal actions. Surveys reveal that mentally ill defendants are judged by community members as lacking free will [18]—mapping onto the volition element of agency. In addition, people believe that mentally ill offenders feel less guilt about their crimes than healthy offenders [29]. This implies that mentally ill offenders are perceived as less able to feel remorse for their crime, potentially because they lack moral knowledge to understand the wrongness of actions—mapping onto the cognitive element of agency.

It is important to note that the link between mental impairment and responsibility need not translate into reduced length of sentencing. A study of over 8,000 criminal defendants who entered a defense of mental impairment from 1976 and 1985 indicated that the defense might actually be associated with a greater likelihood of detainment [30], potentially because people are concerned that these defendants are just finding “loopholes” [31–33]. Alternatively, other people report that mentally ill or impaired defendants should receive a longer term of detainment in order to receive appropriate healthcare to aid recovery [34]. In other words, mentally impaired defendants may be seen as less blameworthy and less deserving of punitive responses, but more in need of help, including potentially detainment.

Situating legal cases within the broader moral psychology literature is helpful not only for understanding the broader mental processes underlying criminal judgments about those using specific legal defenses, but also for making predictions beyond the context of criminal responsibility—here, as will be shortly seen, to lay judgements about offenders’ ongoing societal rights.

### Implications of reduced agency for rights

Not only does mental impairment challenge lay perceptions of responsibility, it may also have downstream consequences for another moral domain: *rights*. Psychological research has shown that perceived mental capacities play a role in lay judgments of rights. The dominant perspective is that perceived agency should have little impact on rights, as moral rights depend on perceptions of the capacity to experience pain (*patiency)*, which is inversely related to agency [20, 35–37]. Under this account, those considered *moral patients* (i.e., entities vulnerable to pain and harm) such as those with a mental impairment, might be seen to deserve *more* rights; in particular, rights pertaining to protection from harm (we expand on this point further below). On the other hand, some recent research suggests that perceptions of perceived agency contribute to judgments of moral status [38]. Here, we advance this debate by examining a more expansive notion of moral rights. We propose that a denial of agency might help mitigate blame, but it may also undermine provision of certain rights.

The aforementioned debate in mind perception and morality has explored a very narrow set of rights—the right to not be harmed [20], or the right to be helped [39]. Following previous nomenclature [40], we refer to those types of rights as *moral standing*. Yet rights are a broad category, including the right to access public goods (e.g., social support services), as well as civil and political rights, such as freedom of speech, the right to privacy, bodily rights, and property rights [41]. Criminal offenders are often subject to diminution of civil rights, such as revocation of one’s right to vote, or prohibitions against hiring people with criminal convictions, even though doing such is not necessarily ordained or codified in sentencing policies [42]. Revocation of these types of rights from criminal offenders has thus been referred to as “invisible punishments” that work as tools of social exclusion [43] by diminishing the rights and privileges normally granted by citizenship and/or residency. Therefore, these types of rights are particularly relevant to lay judgments in the criminal justice context.

When we shift how we conceptualize moral rights beyond the right not to be harmed, the role of moral agency in determining rights becomes more nuanced. All people, from newborn children to the elderly, are typically viewed as deserving certain moral standing [39], such as the right to be treated with care and compassion, or the right not to be harmed. The right to social support services might also be open to anyone, or arguably even more deserving for people who are low in agency and cannot care for themselves. However, only adults capable of higher-order cognition, such as rationality, are given the full legal and political rights to participate in society, including liberties such as the right to vote, to get married, and own property [44]. Such rights are explicitly justified on the basis of agency [45]. The denial of agency in the defense of mental impairment may therefore directly undermine these rights.

### Perceived dangerousness and rights

We predict that a specific aspect of agency, *inhibitive agency*, may play a particularly important role in affordance of rights for defendants with mental impairment. Mentally ill offenders are often perceived as lacking the capacity to inhibit undesirable behaviors (i.e., lacking in inhibitive agency), constituting a threat to community safety [46–48]. Recent research has highlighted that perceived dangerousness is associated with moral standing: people offer less moral protection to entities that is potentially harmful to society [40]. Along these lines, people who are viewed as dangerous due to their lack of agency may thus be attributed less moral standing.

Moreover, treatment in psychiatric facilities (conjuring images of straightjackets and forced medication) may not be seen as rehabilitative by lay audiences, but rather, further stigmatizing [49], potentially due to reinforcement of the belief that a mentally impaired person is unable to control him/herself. The accuracy of these lay perceptions is questionable, as research indicates that perceived recidivism rates for mentally disordered criminals are often overestimated [50]. In fact, defendants who use a mental impairment defense or suffer from psychosis or mood disorders are no more likely—slightly less likely, in some cases—to reoffend than those without these characteristics [51]. Regardless of the accuracy of these judgments, however, people perceived as lacking agency may be stripped of their rights when these judgments are based on perceptions of dangerousness.

Furthermore, lay perceptions of the dangerousness of such defendants are likely to be compounded by the way mental illness is perceived. People tend to view mental illness as biological and constituting a deep part of people’s identity—and therefore as manageable but unlikely to fundamentally change [52]. In line with this, Piazza and colleagues [40] found that it was perceptions of another’s dangerous disposition, rather than another’s actual capacity to act out on that disposition, that was associated with reduced moral standing. That is, mentally impaired people might not only pose an immediate threat to one’s physical safety, but also be perceived to present a more generalized threat to societal order and integrity because of who they are. Thus, the effects of mental impairment on one’s rights may be particularly pernicious.

This topic has significant practical import, because views about mentally ill offenders’ moral status and rights shape public policy. For example, public outrage over a 2016 murder by a man who had previously been under state supervision (following a defense of mental impairment for an earlier crime) led to significant pressure on policy makers to introduce stricter restrictions on those using the defense. While the board responsible for supervisory decisions pushed back on criticisms, stating that such changes “could lead to violations of the rights” of the mentally ill [53], public lobby to diminish rights have in other cases resulted in legislative action [54]. Critically, restoration of the civil rights in particular is essential for those exiting the criminal justice system, given resumption of citizenship roles and inclusion in activities that build social capital are key features of effective rehabilitation [55–57].

### Overview

**Fig 1:**
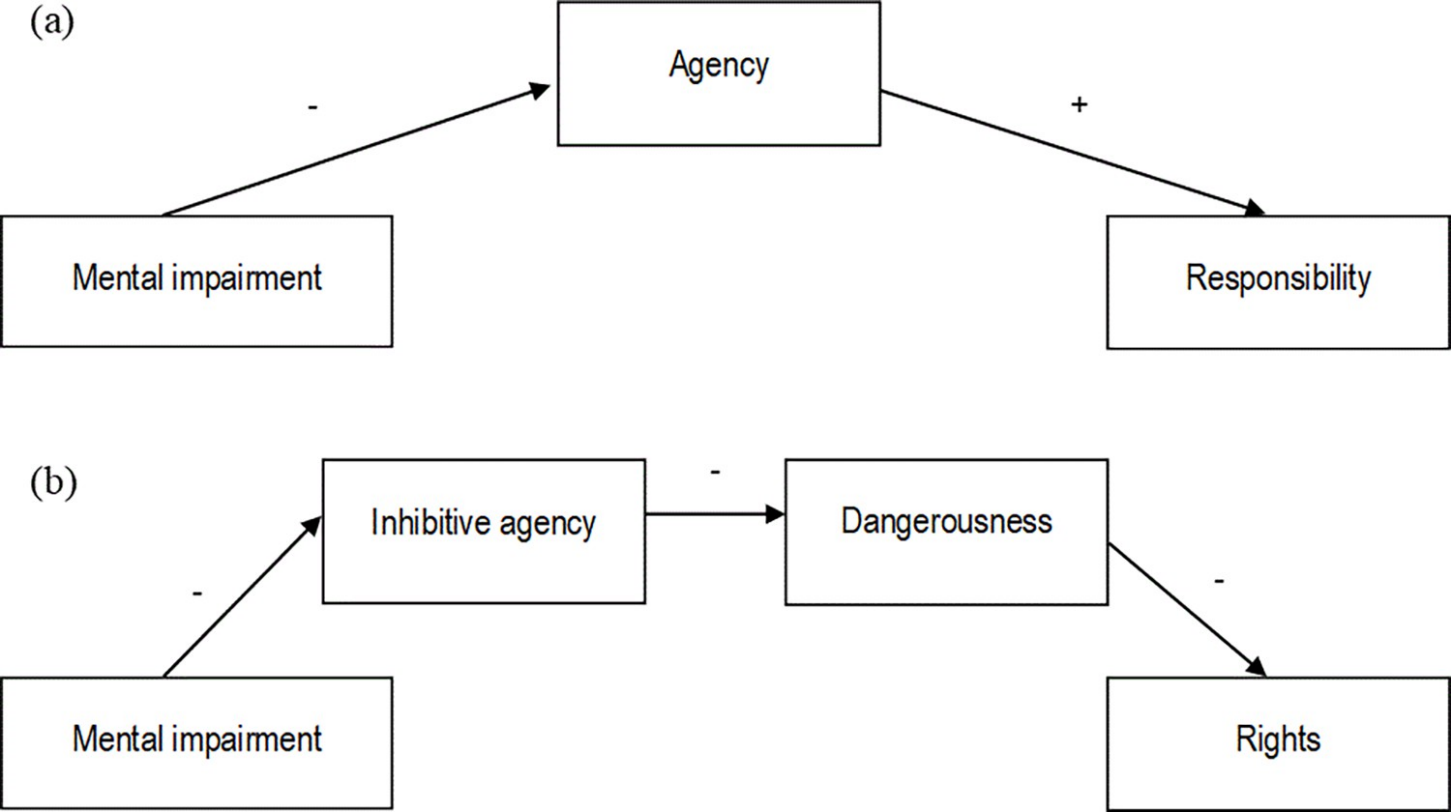
Models of the effects of using the defense of mental impairment on (a) responsibility; and (b) rights.

The present research examines the effects of denial of agency through the defense of mental impairment on judgments of moral responsibility, as well as downstream effects on rights for criminal offenders. Two key models are proposed (see Fig 1): (a) The first model states that a defense of mental impairment will reduce perceptions of the defendant’s agency, which causes reduced attributions of moral responsibility for a criminal act compared to a defendant who plead guilty (Studies 1 and 2); and (b) The second model states that a defense of mental impairment will reduce perceptions of the defendant’s (inhibitive) agency, which increases perceptions of dangerousness and through this, ascription of rights (Studies 1 and 3). Re-establishing agency should counteract these negative effects—leading to less perceived dangerousness and more rights (Study 2). Study 3 (pre-registered) improves on various aspects of the first two studies’ design and more rigorously tests the reliability and credibility of the model pertaining to rights (b).

While mediation analyses cannot establish causality, we contend that attributions of agency are likely to precede judgments of moral responsibility and rights. The causal effects of attributions of mental capacities (including agency and harmful dispositions) on responsibility and moral standing has been established in experimental studies [58], and we manipulate agency directly in Study 2. While we cannot completely rule out the possibility that motivated cognition may account for at least one of the proposed paths (i.e., perceiving entities as more dangerousness as a justification for granting them fewer rights; see [58]), we consider this causal direction less plausible. Nevertheless, the indirect effects reported in this paper should not be interpreted as definitive proof of causal relationships.

Study materials for all studies are presented in the S1 File for this article, and are also publicly available (along with datasets) at https://doi.org/10.17605/OSF.IO/WX543. All three studies reported in this paper were granted ethics approval from institutional review board of the University of North Carolina at Chapel Hill, and all participants provided informed consent to participate via the online survey. All measures, manipulations, and exclusions in the studies are disclosed. Aside from the measures used in this article, we collected additional measures in Studies 1 and 2 that are not central to our analyses. Detail about these measures are provided in the S1 File.

## Study 1

Study 1 provided a preliminary test of our model. We manipulated whether a defendant used the defense of mental impairment (or not) and explored effects on agency, moral responsibility, and rights. The study has two distinct parts, corresponding to the two arms of our model. Specifically, we predicted that (a) using the defense of mental impairment would lead to more lenient crime judgments (less responsibility, blame, and punishment) at trial and that these effects would be mediated by perceived agency. However, after the defendants serve their respective sentences, this mitigation of initial responsibility would come at a cost. Specifically, we predicted that (b) after serving their sentences, non-mentally impaired criminals would be ascribed more rights relative to the newly released mentally impaired patients, and that these differences would be mediated by perceptions of lower agency and increased dangerousness.

### Method

#### Participants

The sample size for Study 1 was primarily determined by budgetary constraints. G*Power 3 [59] indicated that cell sizes of 90 would yield 80% power to detect a medium effect size (*d* = .42) for a two-tailed *t*-test at *p <* .05. We deemed this level of sensitivity acceptable for a preliminary study, noting that past research found medium to large effects of mental disorder manipulations on blame and punishment of offenders [18]. Sample sizes were determined prior to data collection (true for all studies).

Four hundred and twenty-four United States residents completed the survey titled “perceptions of a person” through Amazon Mechanical Turk (MTurk) in exchange for payment. Data integrity measures were used, such as requiring HIT approval ratings over 95%, keeping the task visibility private and including two attention checks (see Materials). Eighty-eight respondents failed attention check items, leaving a final sample of 336 participants (132 males, 200 females, 4 did not indicate gender, *M*_*age*_ = 37.44 years, *SD* = 12.55). We recognize that 88 participants seems like a high number for exclusion, but excluding participants who fail to adequately read instructions is standard practice to ensure data integrity in online research [60].The number of exclusions did not vary significantly by condition, χ^2^ (3) = 1.16, *p* = .763. Including attention check fails produces results consistent with the reported analysis.

#### Procedure

The design was a full between-subjects vignette study design with random allocation to one of four conditions (control at trial, mental impairment at trial, control after release, mental impairment after release). To examine the two different sides of the defense of mental impairment, the study design can be understood as having two distinct separate parts. The first part related to Hypothesis 1 (i.e., that using the defense would lead to more lenient crime judgments). Participants allocated to this part of the study (the trial conditions) received a vignette describing a 25-year-old man (“Jeff”) who was on trial for pushing a stranger off a pedestrian bridge, causing permanent nerve damage and paralysis. Participants read that (a) the defendant pleads guilty (control condition), or (b) the judge rules that Jeff is not guilty by rule of insanity (mental impairment [MI] condition) due to psychotic delusions caused by schizophrenia. To control perceptions of the defendant’s remorse, all participants read that “Jeff is distressed and remorseful about what he has done.” Participants rated the protagonist Jeff on moral responsibility for the offence, along with other judgments related to the crime, as well as agency and dangerousness.

To test Hypothesis 2 (i.e., the long-term negative effects of using the defense on rights), the other half of the sample read a similar vignette about the character several years later, after release from detainment. These participants read the same vignette to begin with, but were given additional information about the defendant’s sentence, in which Jeff was described as having “done his time” and was ready for release, following (a) two years’ detainment in a prison (control condition), or (b) a psychiatric ward (MI condition). Participants then rated the protagonist on agency, dangerousness, and rights.

We used generic vignettes rather than courtroom stimuli for our manipulation because we sought to gauge *lay* attitudes about the psychological and moral dimensions of mentally ill defendants, rather than juror decision-making processes. Structured juror scenarios invoke a legal frame, which introduces cognitive biases in the processing of scientific information [61, 62], making this approach unsuitable for our research question. Here, we are less concerned with mimicking jury processes and more concerned with providing simple information that exposes peoples’ underlying intuitions and beliefs [63].

#### Materials

Unless otherwise indicated, all items were rated on a 5-point rating scale, with scale anchors “Not at all” to “Extremely” (or similar). We report the mean inter-item correlation as a measure of reliability for 3-item scales alongside Cronbach’s alpha, as scales with few items can bias Cronbach’s alpha downwards [64]. A mean correlation above .20 indicates good reliability [65].

*Agency*. We included two measures of agency in this study. First, we constructed a scale of *general moral agency* using traditional concepts of legal responsibility: volition and moral knowledge (scale α = .80). Since the two components of general moral agency were designed to tap into notions underlying legal responsibility for the criminal act itself, we predicted that this type of agency would be most relevant to judgments about the crime (i.e., at trial). We tapped into the volition component with three items (“In general, to what extent is Jeff [willful]; [intentional]”; “To what extent do you think Jeff … can freely choose his own actions”), which were adapted from the battery used in Gray et al. [20]. The scale also included two items relating to moral knowledge (“To what extent do you think Jeff … [knows the difference between right and wrong]; [understands the effects of his behaviour on others]”).

We also measured a second type of agency, *inhibitive agency*, in order to capture agency judgments most relevant to conferral of rights after release form detainment (which we anticipated would function via dangerousness). Three items made up the inhibitive agency scale (“Jeff is able to inhibit negative impulses”; “Jeff would normally refuse to participate in immoral behavior”; “Jeff has the power to refrain from hurting others”), mean inter-item correlation of .24 (α = .50). Inhibitive agency and general moral agency were positively correlated (*r =* .30, *p* < .001).

*Crime judgments*. Participants judging defendants at the trial stage rated the defendant on single-item measures of responsibility (“To what extent is Jeff morally responsible for harming the victim?”), blame (“How much blame does Jeff deserve for his actions?”), and punitiveness (“How much punishment does Jeff deserve for what he did?”). Participants were also asked to indicate how many years Jeff should be detained for (in a prison or psychiatric ward, depending on experimental condition).

*Dangerousness*. A three-item scale assessed dangerousness. We included an item assessing risk of future harm (“How likely do you think it is that Jeff will commit another crime in his lifetime?”), as well as capturing broader judgments of dangerousness (“How dangerous is Jeff currently?”; “How comfortable would you feel attending the same public function as this man?”), mean item intercorrelation of .53 (α = .76).

*Rights*. We included three measures of rights: (1) moral standing, a measure of negative rights consistent with previous research in moral psychology, (2) rights to social services, a measure of an individual’s positive rights to public services, and (3) rights as a citizen. Importantly, rights as a citizen reflect judgments of moral worth and social inclusion, and are not rights that are stripped from those with a mental impairment by sentencing policies; however, they are consistent with notions of liberty and closely tied to many of the invisible punishments historically levied against offenders.

*Moral standing*. Four items assessed moral standing (α = .86), adapted from Piazza et al. [40] and Goodwin and Landy [39], for example, “Harming Jeff would be morally wrong;” “Jeff deserves to be treated with care and compassion”.

*Social services*. Four items measured access to different social services (α = .90), namely whether the state should provide welfare payments, education training, career services and financial advice to Jeff.

*Right as citizen*. Three items measured rights as a citizen (i.e., Jeff’s right to vote, marry, and own property), mean item intercorrelation of .56 (α = .79).

*Attention checks*. To assess whether participants read the vignette properly, a reading check asked participants to select whether the vignette described that Jeff (a) admitted guilt, or (b) was not guilty by reason of mental impairment (though we used the term “insanity defense” throughout the survey to fit the U.S. context). An instructional attention check was also used [60]. Respondents failing these checks (placed at the end of the study) were excluded from the sample.

### Results

In this study, we test for two main predictions: that the defense of mental impairment will reduce responsibility at the time of the trial, and that it will reduce rights after release. Both of these predictions depend on perceptions of agency, which we measured in two different ways. Before turning to each hypothesis, we first report the agency scores and examine how the defense of mental impairment impacts perceptions of moral agency and inhibitive agency both at the time of the trial, and after release.

**Fig 2:**
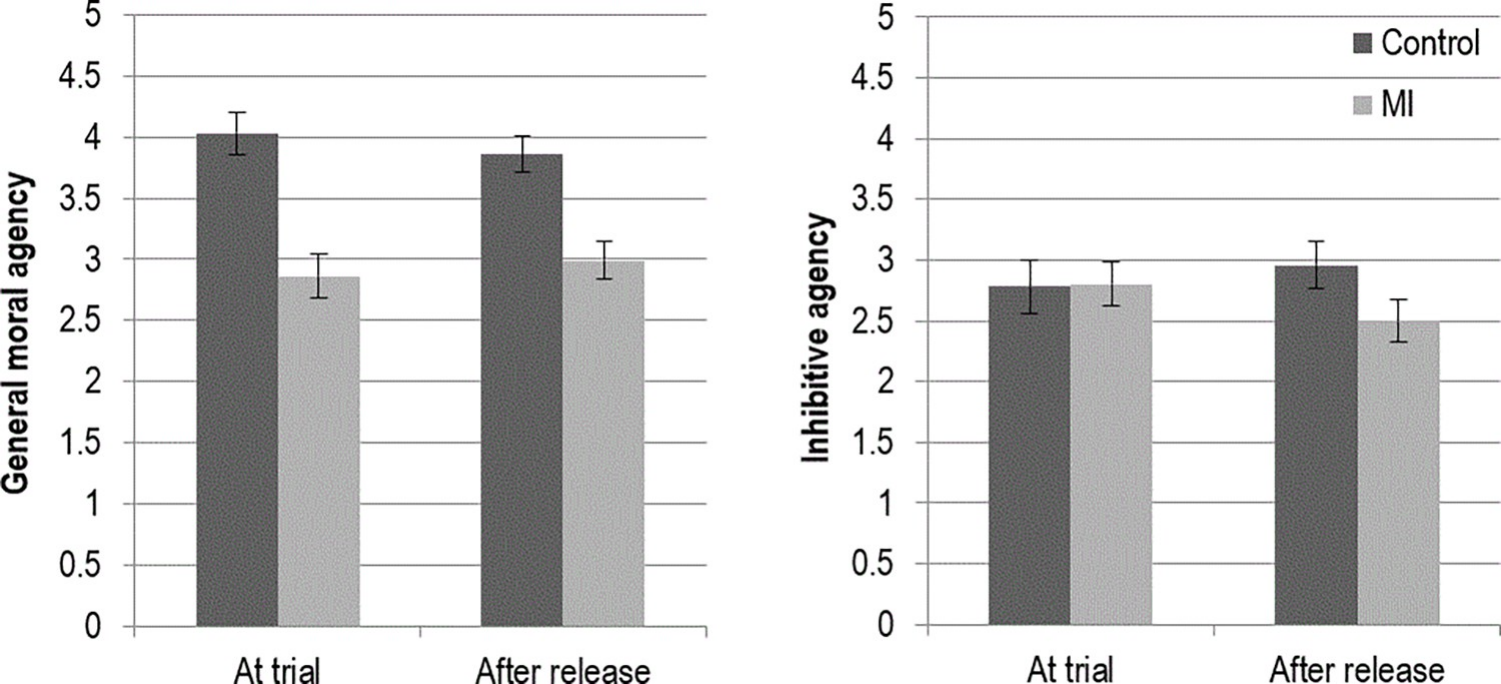
Perceived agency of defendant across experimental conditions (+/- 2 SE).

**Judgments of agency.** Mean scores of the protagonist’s perceived agency across all four conditions are presented in Fig 2. For the analysis of part 1 of the study (comparing control and mental impairment at trial), a *t-*test indicated defendants with mental impairment were perceived to have significantly less moral agency than those in the control condition *t*(166) = 9.33, *p <* .001. There were no differences between the two conditions on inhibitive agency, *t*(166) = -0.15, *p =* .879.

For the analysis of part 2 of the study (comparing control and mental impairment post sentence), mentally impaired defendants were perceived to have less moral agency than control after release from detention, *t*(166) = 8.12, *p<*. 001. MI defendants were also perceived as having significantly less inhibitive agency than control after release *t*(166) = 3.57, *p <* .001.

These results indicate that judgments of general moral agency (rather than inhibitive agency) distinguish mentally impaired defendants from sane defendants at trial. Although not expected, a lack of difference in inhibitive agency at the time of the trial makes sense. Each defendant has just committed a crime that suggests a lack of personal restraint in committing negative acts. Arguably, a more general measure of agency is better able to reliably distinguish responsibility judgments about mentally impaired defendants. After release, however, differences in inhibitive agency were apparent. Mediation analyses conducted in the following sections will examine the critical role of agency in determining crime judgments and in conferring rights to defendants after their release from detainment.

#### Effects of the defense of mental impairment on responsibility at the time of trial

Descriptive statistics for between-group effects for those in the trial groups are presented in Table 1. As predicted, at the time of the trial, using the defense of mental impairment led to less perceived moral responsibility, blame, and deserved punishment for the crime. There were no significant differences between the conditions on length of detainment.

**Table 1:** Descriptive and t-test statistics for dependent variables at trial (Study 1).

	*M* (*SD*)					
Dependent variable	Control (*n* = 86)	MI (*n* = 82)	*t*	df	*p*	*d*
Responsibility	4.86 (0.44)	3.56 (1.23)	9.05	100.45	< .001	1.41
Blame	4.76 (0.59)	3.23 (1.02)	11.75	128.69	< .001	1.84
Punitiveness	4.13 (0.82)	3.11 (0.92)	7.58	166	< .001	1.17
Years detained	11.19 (13.53)	8.26 (10.94)	1.52	164	.129	0.24
General moral agency	4.03 (0.81)	2.86 (0.81)	9.33	166	< .001	1.44
Inhibitive agency	2.78 (1.00)	2.80 (0.82)	0.15	166	.879	0.02

To test whether perceptions of reduced moral agency corresponded to decreases in blame and punishment, we conducted a mediation analysis using general moral agency as a mediator. In order to determine the total, direct, and indirect effects, the PROCESS macro for SPSS (Hayes, 2013; model 4) was used with 10,000 bootstraps and bias-corrected confidence intervals (conservatively adjusted to 99% CIs for multiple tests). As predicted, general moral agency mediated the effect of the defense of mental impairment on judgments of responsibility, blame, and punitiveness (see Table 2). We also tested models using inhibitive agency instead of general moral agency as the mediator. Unsurprisingly, since there was no effect of condition on inhibitive agency, none of the models were significant. Taken overall, the results indicate that participants’ lenient judgments of the mentally ill defendants can be explained—at least partially—by the perception that they lacked moral agency.

**Table 2:** Mediation model statistics: Effects of defense of mental impairment on crime judgments via agency (Study 1).

	M→DV			IV → DV (total effect)			IV → DV (direct effect)			Indirect effect		
Outcome variable	*B*	*SE*	CI_99%_	*B*	*SE*	CI_99%_	*B*	*SE*	CI_99%_	*B*	*SE*	CI_99%_
Responsibility	0.35	.08	0.14, 0.57	-1.30	0.14	-1.67, -0.93	-0.89	0.17	-1.32, -0.46	-0.41	0.11	-0.76, -0.16
Blame	0.42	0.07	0.23, 0.61	-1.52	0.13	-1.86, -1.19	-1.04	0.15	-1.42, -0.66	-0.49	0.10	-0.80, -0.25
Punitiveness	0.29	0.08	0.08, 0.50	-1.02	0.13	-1.37, -0.67	-0.67	0.16	-1.09, -0.26	-0.34	0.10	-0.64, -0.11
Years detained	0.89	1.20	-2.24, 4.03	-2.92	1.92	-7.92, 2.08	-1.88	2.38	-8.09, 4.33	-1.04	1.61	-5.38, 3.25

Condition variable coded as control = 0, MI = 1.

#### Long-term implications of using the defense of mental impairment on rights

Providing some support for our predictions, when Jeff used the defense of mental impairment and had spent time in a psychiatric institution, participants judged him as more dangerous and granted him fewer rights as a citizen, relative to sane Jeff (see Table 3). Participants were, however, *more* likely to endorse access to social services for the mentally ill character, while moral standing did not differ significantly by condition. Thus, results suggest that after serving their time, mentally ill offenders were advantaged in terms of receiving services but they suffered more disadvantage in terms of rights as a citizen.

**Table 3:** Descriptive and t-test statistics for dependent variables after release (Study 1).

	*M* (*SD*)					
Dependent variable	Control (*n* = 87)	MI (*n* = 81)	*t*	df	*p*	*d*
General moral agency	3.86 (0.70)	2.99 (0.69)	8.12	166	< .001	1.25
Inhibitive agency	2.96 (0.89)	2.50 (0.78)	3.56	166	< .001	0.55
Dangerousness	3.83 (0.91)	4.40 (0.88)	-4.11	166	< .001	-0.64
Moral standing	3.81 (1.02)	4.06 (0.87)	-1.66	166	.098	-0.26
Services	2.64 (1.18)	3.05 (1.20)	-2.24	166	.026	-0.34
Rights as citizen	3.89 (0.99)	3.39 (1.14)	3.02	166	.003	0.49

**Fig 3:**
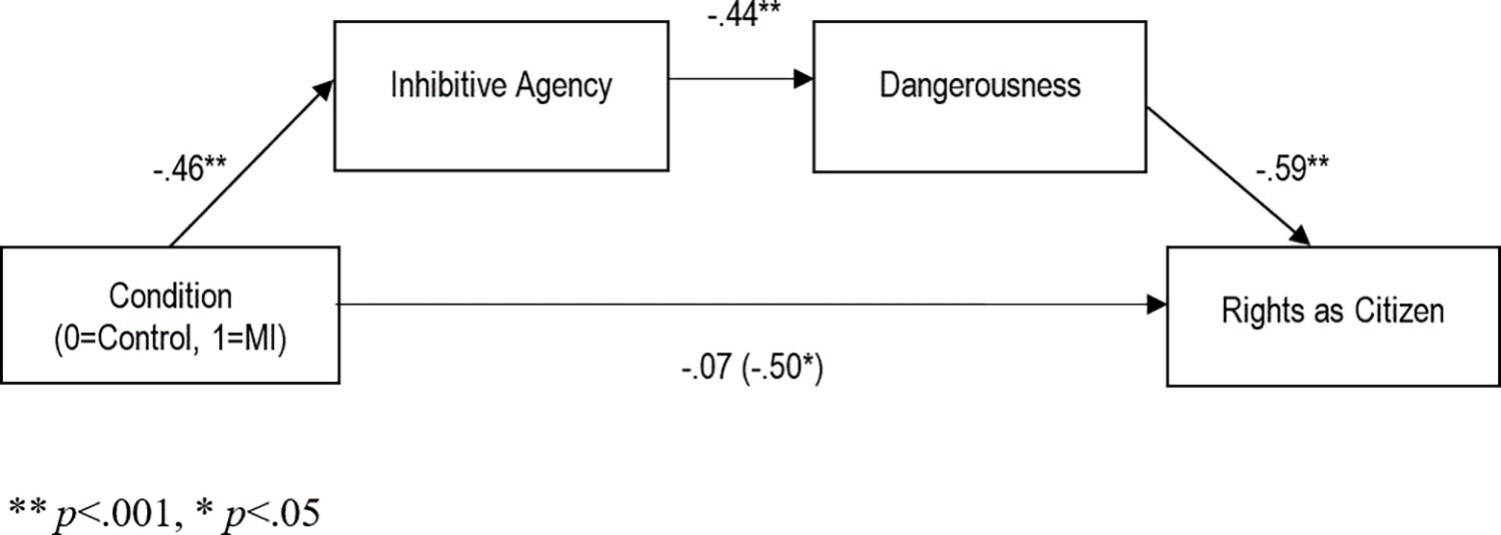
Indirect effect model of using the defense of mental impairment on rights as citizen, via inhibitive agency and dangerousness. Path values are expressed in unstandardized coefficients.

We then examined whether mentally ill Jeff’s reduced rights were related to lower perceived inhibitive agency and higher perceived dangerousness. To test this proposed model (see Fig 3), we conducted a serial mediation analysis (as per Hayes [66] Model 6; 10,000 bootstraps and 99% bias-corrected confidence intervals). The analysis revealed that claiming a defense of mental impairment was associated with fewer rights as a citizen, and that this was mediated by lower levels of inhibitive agency and higher levels of perceived dangerousness, *B*_*indirect*_ = -.12, *SE =* .05, CI_99%_ [-.28, -.03]. This indirect pathway completely mediated the effect of the defense on rights as a citizen (*B*_*direct =*_ -.07, *SE =* .14, *p =* .620).

We also tested whether indirect effects via inhibitive agency and dangerousness could predict scores on the other measures of rights. Despite the direction of the total effect on services being positive (i.e., defense of mental impairment led to more services), the indirect effect was negative, indicating that using the defense had negative consequences on access to social services insofar as participants perceived a loss of inhibitive agency and a subsequent increase in dangerousness (*B*_*indirect =*_ -.09, *SE =* .04, CI_99%_ [-.23, -.02]). In addition, we examined the indirect effect on moral standing, since mediation does not require a total effect of the independent variable on the dependent variable [67]. The indirect effect on moral standing was significant (*B*_*indirect*_ = -.08, *SE* = .04, CI_99%_ [-.22, -.02]) through inhibitive agency and dangerousness. Last, we ran the same mediation models but substituting general moral agency for inhibitive agency; none of the models were significant (*p*s>.05). This indicates that post release, it is not volition or moral knowledge—general moral agency—that matters for rights (indeed, lacking these capacities, such persons may simply be passive and accepted members of society), but rather, the ability to control the harm one may inflict on society in the future.

### Study 2

Study 1 demonstrated that while the defense of mental impairment can reduce perceived responsibility for a crime, the lack of agency implied by one’s mental illness also reduces certain rights in the long term. Yet, it is possible that the effects observed in Study 1 were not due to reduced agency per se, but due to other variables commonly associated with mental illness, such as generalised stigma and disgust [68, 69].

Study 2 was designed to provide a more robust test of the causal and critical role of agency in reduced moral status for those using a defense of mental impairment. We replicated and extended the experimental design in Study 1 with a key change: we included *two* mentally impaired (MI) conditions at the post-sentence (after release) level, in additional to the control condition. Here, we directly manipulated inhibitive agency (the proposed mediator for the effects of the defense of mental impairment on rights) by describing the mentally impaired offender as having regained inhibitive agency (versus not). This design thus employs a *blockage* manipulation [70] to block the proposed mediation process. Though in both of the mental impairment conditions, Jeff is still mentally ill—thereby controlling for mental illness stigma (and stereotypes pertaining to his mental illness in particular)—we varied only the extent to which he had learned to manage his illness and control his behavior (i.e., the extent to which he had regained inhibitive agency). This is consistent with law in many jurisdictions, where forensic patients can be held indefinitely until they have demonstrated such agency (at which time they may be released). If agency is a mediator of the defense, then reduced levels of rights should be observed when Jeff has not learned to control his impulses (MI-unrecovered condition), but not when he has learned to do so (i.e., when he has regained his agency; MI-recovered condition).

We expected to replicate results of Study 1 in relation to implications at trial: mental impairment would reduce perceived responsibility, blame, and punitiveness, and this would be mediated by moral agency. With regard to the second arm of the model, we expected those released from a psychiatric ward and unable to control their impulses would be viewed as lacking in inhibitive agency, more dangerous, and having fewer rights relative to those released from prison. We predicted that this diminution in rights would be across the board; that is, we would observe a consistent negative (total) effect across all three rights measures. While there was some evidence in Study 1 that the defense might increase access to services, the lack of inhibitive agency (and therefore dangerousness) of the mentally impaired (unrecovered) character will be more salient, and therefore we expected negative effects of agency on rights to be stronger. Further, we predicted that regaining inhibitive agency would nullify any negative effects of the defense of mental impairment on later rights, such that using the defense is not a barrier to social reintegration so long as one’s agency is regained (mediated by a reduction in perceived dangerousness).

### Method

#### Participants

We slightly increased our desired sample size relative to Study 1, as we reasoned that mean differences between the two post-sentence mental impairment conditions (i.e., recovered versus unrecovered agency) could be slightly smaller than those between the control condition and the unrecovered agency condition (i.e., equivalent to the control–MI contrast in Study 1). Working from the main effect of condition on rights as citizen of *d =* 0.49 in Study 1, we chose a *d* of 0.40 as the smallest effect size of interest in Study 2 if the recovered agency condition is to be meaningfully distinguished from the unrecovered agency condition. G*Power indicated a sample of 100 per condition was required for 80% power to detect one-tailed contrasts in the predicted directions between two independent groups of *d =* 0.40 at *p =* .025 (adjusted for multiple comparisons between the three post-sentence conditions).

Seven hundred and sixty-seven United States residents completed the survey through MTurk. One hundred and eighty-six respondents were excluded for failing the attention checks. Including attention check fails produces results consistent with the reported analysis. The final sample was 581 participants *(M*_*age =*_ 39.05 years, *SD =* 13.29; 57% female, 43% male).

#### Procedure

Study 2 used a design nearly identical to Study 1, with the exception that after release, we included two mental impairment conditions (in addition to control), which allowed us to empirically test whether it was an ongoing reduction in inhibitive agency, and a corresponding increase in perceived dangerousness, that accounted for any reduction in rights upon release from detainment. Participants in all three post-sentence conditions (including the control condition) read that before release, Jeff had a hearing to review his progress, in which the wardens (or prison officers, in the case of the control condition) and psychologists at the facility in which Jeff was detained testify that “Jeff is eligible for release” and is therefore returned to society, having “done his time.” The two MI conditions received some additional information. In the MI-recovered condition, participants read that “they state that Jeff is in control of his illness and has learned to manage his illness independently. They also testify that Jeff has developed the capacity to control his emotions and impulsive behaviors.” In contrast, in the MI-unrecovered condition, participants read, “he could, however, learn to be more in control of his illness and to better manage his illness independently. They also testify that Jeff has struggled at times to control his emotions and impulsive behaviors”.

#### Materials

Measures were unchanged from Study 1, with the following exceptions. We sought to improve the internal reliability of the three-item inhibitive agency scale by amending one item (that had weak correlations with the other items) to be more consistent with the others: “Jeff has the capacity to resist doing the wrong thing” (scale mean item intercorrelation = .81, α = .93). In addition, we modified the six-item general agency scale. Two of the scale items were changed to increase the specificity and face validity of the terms (“intentional” and “willful” were replaced with “mental capacity to think about and plan for the future” and “exercise self-control”), and an extra item measuring the moral knowledge dimension of the scale was added (“is aware of social norms”) to balance it with the volition dimension. Internal consistency for the general agency scale was excellent (α = .91).

We focused on different measures of agency across the conditions. Since Study 1 results confirmed that general moral agency was relevant to judgments about the crime (responsibility, blame, and punitiveness), the general moral agency scale was only presented to participants in the trial condition. For the post-sentence analysis we only measured judgments of inhibitive agency, since Study 1 indicated that this measure was more relevant for conferring rights.

Remaining scales showed good internal reliability: dangerousness (mean item intercorrelation = .56, α = .79), moral standing (α = .82), access to social services (α = .89), rights as citizen (mean item intercorrelation = .62, α = .82).

Along with the two attention check items from Study 1 (presented to all participants), we presented an additional attention check item to those in the MI conditions post sentence, in order to eliminate those who did not read the recovery manipulation vignette properly. Participants were asked whether in the vignette, wardens and psychologists testified at Jeff’s hearing that Jeff had: (a) learned to control his illness and impulses, or (b) struggled to control his illness and impulses. Those who answered incorrectly were excluded from the analyses.

## Results

### Effects of the defense of mental impairment on responsibility at the time of trial

Replicating Study 1, at the time of trial, using the defense of mental impairment led to less moral responsibility, blame, and punishment for harming the victim (see Table 4). There were no differences in the length of time participants believed the perpetrator should be detained.

**Table 4:** Descriptive and t-test statistics for dependent variables at trial (Study 2).

	*M* (*SD*)					
Dependent variable	Control (*n* = 125)	MI (*n* = 123)	*t*	df	*p*	*d*
Responsibility	4.71 (0.66)	3.52 (1.22)	9.57	186.99	< .001	1.21
Blame	4.63 (0.76)	3.42 (1.17)	9.63	208.06	< .001	1.23
Punitiveness	4.15 (0.80)	3.24 (1.10)	7.43	223.61	< .001	0.94
Years detained	13.47 (13.61)	15.55 (22.99)	-0.85	185.91	.391	-0.11
General moral agency	3.56 (1.05)	2.75 (0.99)	6.23	246	< .001	0.79

Next, we examined the predicted model of effects on crime judgments via moral agency. Mediation models were tested as per the procedure detailed in Study 1. Results indicated that using the defense of mental impairment led to less perceived responsibility, blame, and punitiveness (but not years detained), partially through a reduction in perceived moral agency (see Table 5).

**Table 5:** Mediation model statistics: Effects of defense of mental impairment on crime judgments via agency (Study 2).

	M→DV			IV → DV (total effect)			IV → DV (direct effect)			Indirect effect		
Outcome variable	*B*	*SE*	CI_99%_	*B*	*SE*	CI_99%_	*B*	*SE*	CI_99%_	*B*	*SE*	CI_99%_
Responsibility	0.22	0.06	0.07, 0.38	-1.19	0.12	-1.51, -0.87	-1.01	0.13	-1.35, -0.67	-0.18	0.06	-0.37, -0.06
Blame	0.21	0.06	0.05, 0.37	-1.21	0.13	-1.53, -0.88	-1.04	0.13	-1.38, -0.70	-0.17	0.06	-0.36, -0.04
Punitiveness	0.21	0.06	0.06, 0.36	-0.91	0.12	-1.22, -0.59	-0.74	0.13	-1.07, -0.41	-0.17	0.06	-0.36, -0.04
Years detained	0.86	1.17	-2.19, 3.91	2.07	2.41	-4.19, 8.33	2.76	2.59	-3.97, 9.49	-0.69	1.03	-3.36, 2.29

Condition variable coded as control = 0, MI = 1.

#### Long-term implications of using the defense of mental impairment on rights: Mentally ill offenders can be redeemed—if they regain agency

Next we explored the implications of the defense for perceived rights: whether lack of agency hindered mentally ill offenders from being accepted back into society post-release, and whether these effects could be mitigated by recovered agency. See Table 6 for group means on all dependent variables after perpetrator release. The below analyses draw on one-way ANOVA models, examining contrasts between the three post-sentence conditions.

**Table 6:** Descriptive and inferential statistics for dependent variables after release (Study 2).

	*M (SD)*					
Dependent variable	Control (*n* = 115)	MI recovered (*n* = 115)	MI unrecovered (*n* = 103)	*F*	df	*p*
Inhibitive agency	3.55 (1.02)^a^	3.42 (0.93)^b^	2.70 (0.95)	24.22	2, 330	< .001
Dangerousness	3.62 (0.89)^a^	3.78 (0.84)^b^	4.37 (0.83)	22.95	2, 330	< .001
Moral standing	4.01 (0.76)	4.19 (0.74)	3.97 (0.86)	2.61	2, 330	.08
Services	2.76 (1.27)	3.01 (1.12)	2.79 (1.21)	1.42	2, 330	.243
Rights as citizen	3.93 (0.99)^a^	3.89 (0.97)^b^	3.48 (1.14)	6.20	2, 330	.002

^a^Control different to MI unrecovered (*p* < .05).

^b^MI recovered different to MI unrecovered (*p* < .05).

Analyses revealed that after release, participants attributed more inhibitive agency to sane Jeff (control) than to unrecovered Jeff. However, attributions of inhibitive agency did not differ between control and the recovered agency condition. This suggests that a defendant described as having learned to regain control of their behavior was perceived as having equal inhibitive agency to a defendant without a mental illness. Comparing the two mental impairment conditions confirmed that recovered Jeff was perceived as possessing significantly more inhibitive agency than unrecovered Jeff—as would be expected if the manipulation were successful.

**Fig 4:**
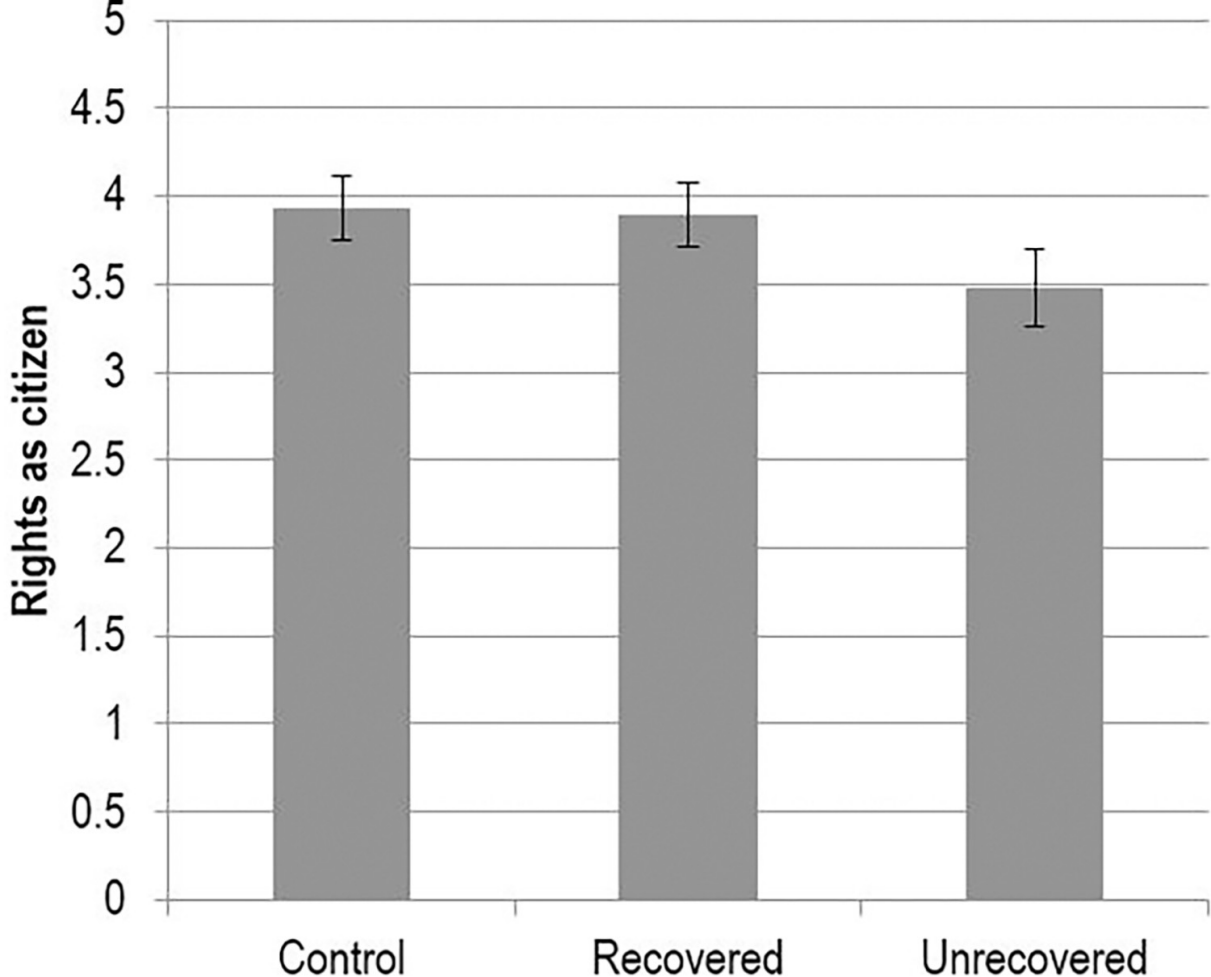
Means scores for rights as citizen measure (+/- 2 SE).

In line with predictions, when Jeff had used the defense of mental impairment and had not recovered any agency, participants found him to be more dangerous, and granted him fewer rights as a citizen, relative to sane Jeff. They did not, however, grant him fewer services or less moral standing, going against our expectations. In contrast, when Jeff had used the defense and had recovered his agency, no differences existed at all; he was treated in the same manner as his sane counterpart across all outcome variables. In addition, recovered Jeff fared significantly better than unrecovered Jeff in terms of perceived dangerousness and rights as a citizen (e.g., see Fig 4). Overall, the results supported our hypothesis: Recovering one’s agency nullified the deleterious effect of using the defense of mental impairment on dangerousness and rights.

Finally, we tested whether the restoration of inhibitive agency was responsible for the differences observed between the MI-recovered and MI-unrecovered conditions. Since inhibitive agency was directly manipulated in Study 2, using the same serial mediation model as in Study 1 (condition → inhibitive agency → dangerousness → Y) would be problematic—the exogenous variable (restoration of inhibitive agency) and the mediator (inhibitive agency) are too proximal and would produce multicollinearity [71]. Instead, we omitted the measured inhibitive agency scale and conducted a simple mediation model (condition → dangerousness → Y), with the contrast between unrecovered and recovered conditions set as the exogenous variable (unrecovered = 0, recovered = 1) [72].

The analysis revealed that dangerousness completely mediated the effect of the agency manipulation on rights as citizen. In addition, there were significant indirect effects on services and moral standing in line with our model (see Table 7). These results indicate that when those using the defense of mental impairment are seen as less in control of their actions, they are perceived as more dangerous, and this corresponds to being granted fewer rights of all kinds.

**Table 7:** Mediation model statistics: Effects of recovered agency on rights via dangerousness (Study 2).

	M→DV			IV → DV (total effect)			IV → DV (direct effect)			Indirect effect		
Outcome variable	*B*	*SE*	CI_99%_	*B*	*SE*	CI_99%_	*B*	*SE*	CI_99%_	*B*	*SE*	CI_99%_
Moral standing	-0.36	0.06	-0.51, -0.20	0.23	0.11	-0.05, 0.51	0.02	0.11	-0.26, 0.29	0.21	0.06	0.09, 0.40
Services	-0.45	0.09	-0.68, -0.21	0.21	0.16	-0.20, 0.62	-0.05	0.16	-0.47, 0.36	0.26	0.08	0.10, 0.53
Rights as citizen	-0.63	0.07	-0.82, -0.43	0.41	0.14	0.04, 0.78	0.04	0.13	-0.30, 0.38	0.37	0.09	0.17, 0.65

*Note*. Condition variable coded as MI unrecovered = 0, MI recovered = 1.

## Study 3 (registered study)

Study 2 confirmed that reducing agency can reduce responsibility for one’s crime, but has negative ramifications for rights. Moreover, it showed that inhibitive agency plays a causal role in conferring rights, especially as it relates to dangerousness. When the mentally impaired defendant was able to demonstrate he had regained his inhibitive agency, he was able to avoid the deleterious effects of the defense of mental impairment.

Taken together, the results of the first two studies suggest that laypeople judge those using a mental impairment defense as not only less responsible for their actions and thus their crime, but possessing less inhibitive agency—and consequently being less deserving of certain rights. When we examined the most traditional definition of rights (moral standing), we encountered mixed findings. The defense of mental impairment increased (Study 1) or had no effect on (Study 2) moral standing at release relative to a guilty plea, though the indirect effect of reduced agency on moral standing via dangerousness was negative in both studies. It is possible that the defense exerts heterogenous effects on rights, involving both positive and negative indirect effects. Specifically, the mental impairment defense might signal increased *patiency* as well as diminished agency—which would theoretically increase rights, particularly those that are strongly tied to notions of vulnerability. Moral standing represents rights that have typically been associated with entities requiring protection (moral patients), so it is not surprising that those with mental impairment could be granted these rights (as in Study 1). Where one’s lack of agency is made particularly salient (as in the unrecovered MI condition in Study 2), the negative effects via diminished agency may override any positive effects via patiency on rights.

Moving beyond the basic negative rights definition involving protection from harm, we also examined *positive* liberties, such as the right to marry, as well as the defendant’s rights to social services. Access to services followed the pattern seen for moral standing—the mental illness defense either increased access to services (Study 1) or did little to affect it (Study 2), though there was a consistent negative indirect effect of the defense (or manipulated agency in Study 2) via dangerousness. Again, access to services might imply vulnerability to some extent, possibly leading people to grant those using the defense more of these rights.

Rights as citizens showed a more consistent pattern in relation to agency—both total effects and indirect effects across both studies—likely because these rights more naturally align with moral agency, as rights of citizenship assume adult agency. This may also be because citizenship rights are most pertinent to the criminal justice realm, reflecting our historical tendency to socially exclude offenders through diminution of such rights [42, 43].

We included an exploratory patiency measure in Study 1, which allowed us to test the idea about a second, opposing, indirect effect through patiency. These data provided support for this hypothesis (results are presented in the S1 File). However, since this analysis was based on post-hoc reasoning, a confirmatory, pre-registered study was necessary to test for these two indirect effects in a more robust fashion.

Our first two studies have some limitations. Most notably, we only used one example of a crime and mental impairment. The effect may not generalize to other circumstances, even though we theoretically expect it to. For instance, according to our framework, any reduction in agency should yield similar effects, regardless of type of mental illness.

The primary aim of the pre-registered Study 3 was to conduct a conceptual replication of Study 1, focusing on the more novel arm of the research—Hypothesis 2: that the defense of mental impairment corresponds with reduced rights at the end of a sentence. That is, the study was a 1-factor, 2-level (defense vs. control) experimental vignette design. We tested for heterogenous effects of the defense on rights, exploring two indirect effects of the defense on rights (that is, one via agency/dangerousness and one via patiency). Study 3 thus offered the opportunity to better explain positive (or null) main effects of the manipulation on rights. In addition, the pre-registered study addressed concerns regarding the generalizability of the research by using a stimulus sampling approach. In this design, participants were exposed to multiple vignettes varying on other (theoretically irrelevant) features. If the proposed effect is robust, it should hold across differing stimuli. The registered study thus aimed to increase confidence in the model and earlier findings. The study protocol was accepted by PLOS ONE [73].

### Hypotheses

Study 3 tested five hypotheses.

Hypothesis 1: Mentally impaired defendants would have less inhibitive agency, and would be perceived as more dangerous than control.

We predicted differentiated main effects of the defense on rights. Since rights as a citizen are more closely tied to agency, we still expected to observe a negative main effect of the defense on this measure. That is:

Hypothesis 2: Those using the defense would be deemed less deserving of rights as a citizen relative to control.

In contrast, we predicted that the defense would result in *more* (or no change in) moral standing and access to services. That is:

Hypothesis 3: Those using the defense would be granted more (or equal) moral standing and right to social services relative to control.

Based on the results of the first two studies, there is reason to suspect that an alternate mediator is exerting a counteracting influence on rights. We therefore expected to find two indirect effects of the defense on these all three rights measures. This constitutes the fourth hypothesis:

Hypothesis 4: We would observe a negative indirect effect on rights via inhibitive agency and dangerousness; and a positive indirect effect via patiency.

We made further predictions about the nature of these indirect effects in the final hypothesis:

Hypothesis 5: We expected the indirect effect on rights as citizen via the inhibitive agency and dangerousness route to be stronger than the indirect effect through the patiency route. In contrast, in the models for access to services and moral standing, we expected the indirect effect via patiency to be equal in magnitude to the indirect effect via inhibitive agency and dangerousness.

### Method

#### A priori power analysis

We determined that the sample should have enough statistical power not only to detect medium and large (main) effects, but that smaller differences between control and MI—such as those consistent with effects observed on moral standing (*d* = 0.26) and access to services (*d* = 0.34) in Study 1—would also be meaningful. We therefore set our smallest effect size of interest to a small-medium *d* of 0.3. According to G*Power, in order to obtain 90% power to detect an effect of this magnitude using a one-tailed independent samples *t*-test (with alpha set at .0167 to adjust for testing of three rights measures) we require 260 participants per condition.

We followed the method and software described in Schoemann et al. [74] to calculate statistical power required for the indirect effects. Using Monte Carlo simulations and the exact parameters from Study 1, the analysis indicated that an *N* of 520 would achieve a power >99% (at α = .0167 to correct for mediation analyses on three rights measures) to detect the indirect effect of condition on moral standing via inhibitive agency and dangerousness—the smallest indirect effect of condition on any rights measure in that study. Since any counteracting mediation via patiency was expected to be similar (or larger) in magnitude to the negative effect via inhibitive agency and dangerousness, this power analysis is suitable for testing both pathways.

#### Participants

United States residents were recruited from MTurk. As per the two preliminary studies, exclusion criteria were limited to MTurk users with a HIT approval rating below 95% and respondents failing attention checks. No interim analyses or stopping rules were applied to data collection.

The initial sample consisted of 703 respondents. Only 19 participants were excluded for failing attention and manipulation checks (see below), thus the final sample consisted of 684 participants (*M*_*age*_ = 41.86, *SD* = 12.41; 327 female, 352 male, 5 non-binary)—slightly larger than planned. The sample was fairly politically diverse, with 52.78% of participants identifying as politically liberal, 29.09% as conservative, and 18.13% as moderate (*M*_*political orientation*_ = 3.48, *SD* = 1.81).

#### Exclusion criteria

Data were screened to exclude participants who failed the attention check, which involved responding to an item placed at the end of the survey, identical to the one in Study 1 (asking whether the character was found guilty or not guilty by reason of insanity). Any respondent who did not check the correct response was excluded from further analysis. In addition, an instructional attention check was placed at the beginning of the survey, filtering out respondents before they could complete the study. This latter group were not considered dropouts.

#### Materials

The experimental manipulation of the defense of mental impairment was conceptually similar to the one used in Study 1, except for varying features of the vignette. Participants read one of several vignettes describing a person (Liam) who has recently completed the terms of their court order. The character was described as having committed a crime several years early and was either: found guilty of the crime (control condition); or found not guilty by mental impairment (mental impairment condition). Vignette features varied by: type of crime (robbery or sexual offending); type of sentence (not crossed but matched to the crime; custodial- or community-based sentence/treatment); and type of mental impairment (bipolar disorder or brain injury). Crossing of vignette features resulted in six unique vignettes. These are presented in the S1 File along with outcome measures.

Outcome measures were comprised of inhibitive agency (α = .89), dangerousness (α = .78), and rights measures (moral standing [α = .83], rights to social services [α = .89], and rights as a citizen [α = .76]). These were unchanged from Study 2. We also introduced a three-item patiency measure (α =. 92; Goya-Tocchetto, Gray, Vaisey, & Kapsaskis, 2019, unpublished manuscript).

### Analysis plan

Independent samples *t*-tests were used to test for differences between the control and MI conditions on inhibitive agency, dangerousness, and rights measures (Hypotheses 1, 2, and 3). We tested Hypothesis 1 (mentally impaired defendants would have less inhibitive agency, and would be perceived as more dangerous than control) and Hypothesis 2 (mentally impaired defendants would be deemed less deserving of rights as a citizen relative to control) using a one-tailed test. We tested Hypothesis 3 (mentally impaired defendants would be granted more, or equal, moral standing and right to social services) using a two-tailed test. For the tests on rights, an alpha correction was applied (α = .017) to account for the inclusion of three rights measures.

To examine indirect effects (Hypotheses 4 and 5), three mediation analyses were conducted—one for each rights measure as a dependent variable—using SEM. While we report model fit indices, they are not important for testing our predictions, which pertain to the indirect pathways. Specifically, use of the mental impairment defense would lead to less inhibitive agency, which would be associated with an increase in dangerousness and a corresponding diminution of rights. At the same time, a counteracting pathway to rights would be observed whereby the defense increases patiency and rights. The alpha level for each indirect effect was set to .017 to adjust for the use of three rights measures.

### Results

Descriptive and inferential statistics comparing the two experimental conditions on outcome variables are presented in Table 8. As predicted (Hypothesis 1), participants in the MI condition perceived the defendant as significantly lower in inhibitive agency, and significantly more dangerous, than participants in the control condition. We also observed that defendants in the MI condition were granted more patiency than control.

**Table 8:** Descriptive and t-test statistics for dependent variables (Study 3).

	*M* (*SD*)					
Dependent variable	Control (*n* = 352)	MI (*n* = 332)	*t*	df	*p*	*d*
Inhibitive agency	3.52 (1.07)	3.07 (0.74)	5.89	679	< .001	0.45
Dangerousness	2.94 (0.75)	3.18 (0.89)	-3.60	682	< .001	–0.28
Patiency	3.04 (0.92)	3.35 (0.95)	-4.46	682	< .001	–0.34
Rights	4.41 (0.76)	4.16 (0.93)	3.87	682	< .001	0.30
Moral standing	4.10 (0.81)	4.45 (0.80)	-2.37	638	.001	–0.18
Services	3.02 (1.20)	3.31 (1.15)	-3.22	682	.018	–0.25

Turning to the rights measures, in line with predictions, those using the MI defense were deemed less deserving of rights as a citizen (Hypothesis 2) but more or just as deserving of moral standing and services (Hypothesis 3), relative to control.

We then examined whether the effect of the MI defense on rights could be accounted for by (a) a negative indirect effect via inhibitive agency and dangerousness, and (b) a positive indirect effect via patiency (Hypothesis 4). Further, we had predicted that the indirect effect via inhibitive agency and dangerousness would be stronger than the indirect effect via patiency for rights as a citizen, but that the indirect effects would be equal in magnitude for moral standing and access to services (Hypothesis 5). To test these predictions, we ran a series of three path analyses using AMOS (v. 26) examining each of the three measures of rights. Path coefficients were calculated using maximum likelihood estimation and 98.3% bias correct bootstrapped standard errors and confidence intervals (5000 resamples).

**Fig 5:**
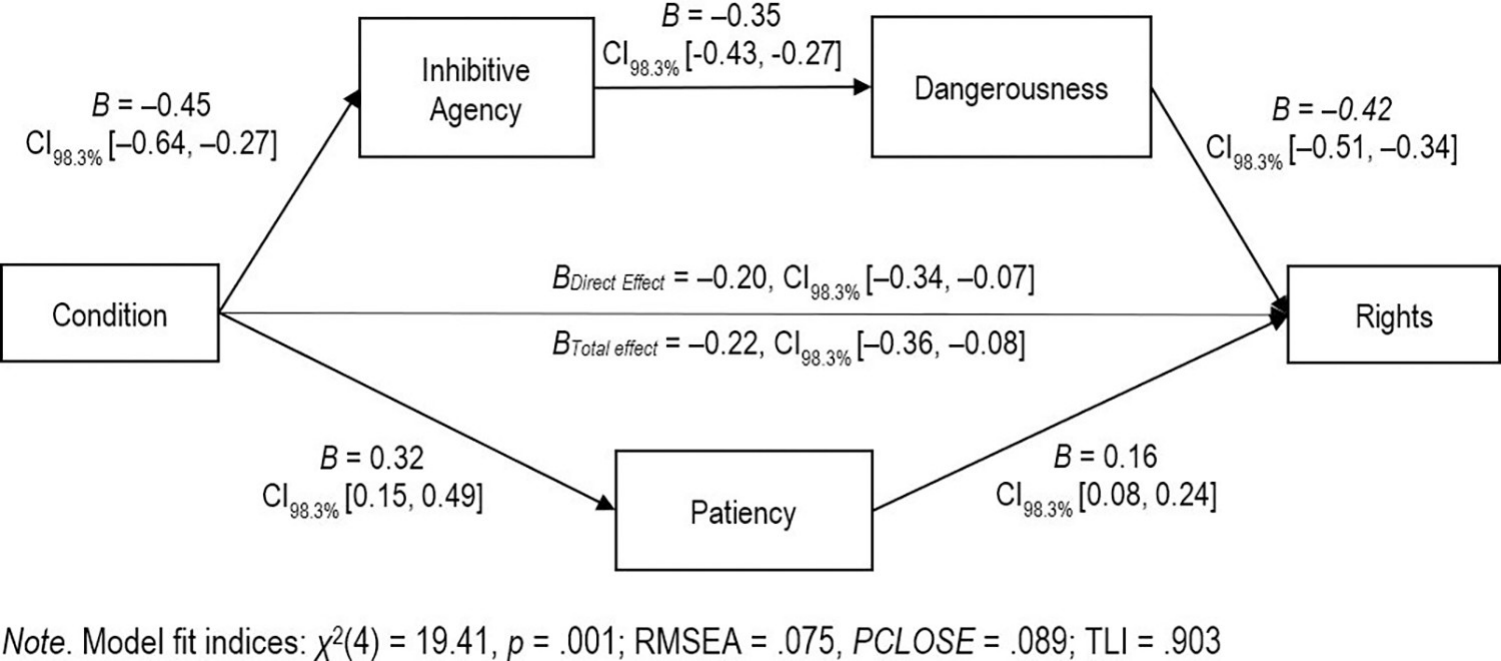
Dual path model of using the defense of mental impairment on rights as a citizen. Path values are expressed in unstandardized coefficients.

Results supported our hypotheses for rights as citizen. Regression coefficients are presented in Fig 5. There were negative, significant direct and total effects of MI condition on rights as a citizen. In line with predictions, there was also a negative indirect effect of condition via inhibitive agency and perceived dangerousness (*B* = –0.07, CI_98.3%_ [–0.10, –0.04]) as well as a positive indirect effect via patiency (*B* = 0.05, CI_98.3%_ [0.02, 0.10]). The magnitude of the standardized indirect effect via inhibitive agency and dangerousness (*β* = 0.09) was significantly greater than the magnitude of the indirect effect via patiency, (*β* = 0.03; *B*_*diff*_ = 0.12, CI_98.3%_ [0.08, 0.17]).

**Fig 6:**
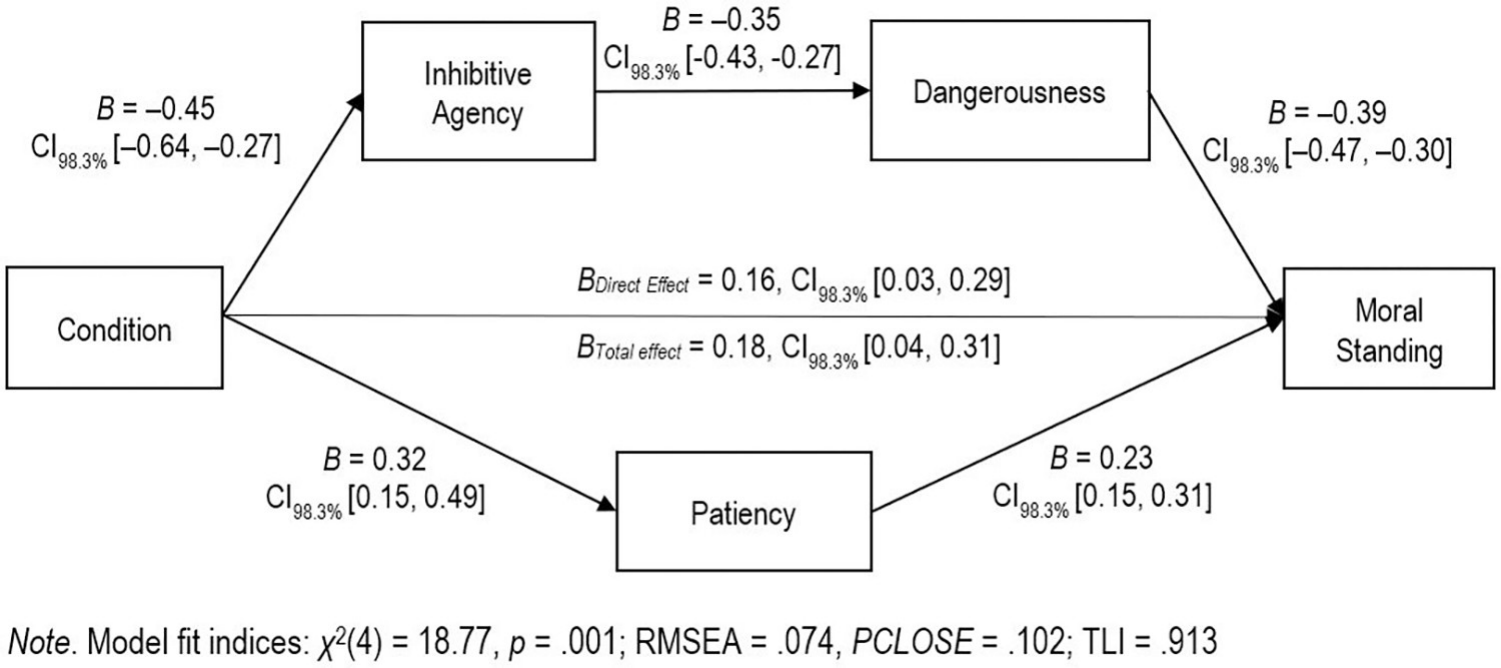
Dual path model of using the defense of mental impairment on moral standing. Path values are expressed in unstandardized coefficients.

Though we had expected the pattern of effects on moral standing and access to services to be slightly different to those for rights as citizen (i.e., we expected the indirect effect through patiency to be larger than the other indirect pathway via inhibitive agency and dangerousness), similar patterns to rights were observed. The analysis revealed significant, positive direct effect and total effects of MI condition on moral standing (see Fig 6 for unstandardized coefficients). In line with predictions, there was a negative indirect effect via inhibitive agency and dangerousness (*B* = –0.06, CI_98.3%_ [–0.10,– 0.04]) and a positive indirect effect of condition on moral standing via patiency (*B* = 0.07, CI_98.3%_ [0.03, 0.13]). The magnitude of the indirect effect via inhibitive agency and dangerousness (*β* = 0.09) was significantly larger than the magnitude of the indirect effect via patiency, (*β* = 0.05; *B*_*diff*_ = 0.14, CI_98.3%_ 0.09, 0.20).

**Fig 7:**
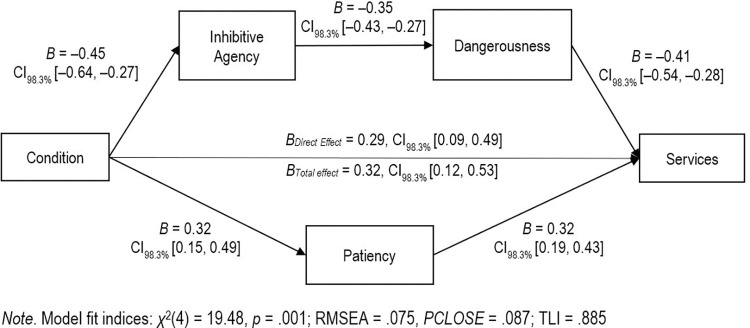
Dual path model of using the defense of mental impairment on access to services. Path values are expressed in unstandardized coefficients.

For access to services, there were positive direct and total effects of MI condition on access to services (see Fig 7 for unstandardized coefficients). Critically, there was again a negative indirect effect via inhibitive agency and dangerousness (*B* = –0.07, CI_98.3%_ [–0.11, –0.04]) and a positive indirect effect on services via patiency, (*B* = 0.10, CI_98.3%_ [0.05, 0.18]). The magnitude of the standardised indirect effect via inhibitive agency and dangerousness (*β* = 0.09) was significantly larger than the magnitude of the indirect effect via patiency (*β* = 0.04; *B*_*diff*_ = – 0.17, CI_98.3%_ [–0.25, –0.10]).

Across all three models, individual paths were consistent with our model: MI condition negatively predicted inhibitive agency, which in turn negatively predicted dangerousness, which negatively predicted rights. MI condition positively predicted paitency, which in turn positively predicted rights. However, the finding that the inhibitive agency and dangerousness pathway was consistently larger than the pathway via patiency indicates that the perceived threat of future danger posed by mentally impaired defendants outweighs their perceived need to be helped.

We also conducted exploratory analyses examining differences between mental impairment types and crime types (see S1 File).

## Discussion

In the legal system, the defense of mental impairment confers reduced criminal responsibility to those who, for reason of mental impairment, lack the ability to fully comprehend and/or control their behavior [3, 5]. Consistent with previous research [10–15, 17–19], our results demonstrate that these legal principles are echoed in judgments made by members of the general population. In addition, we demonstrate the critical (and double-edged) role of agency in underpinning such perceptions. Our findings indicate that, in denying agency, a defense of mental impairment may protect mentally ill defendants from a legal perspective; however, this can also limit the extent to which such defendants are granted a meaningful return to society in the longer term. Our findings were found to be robust across different types of mental impairment, offences/sentences, and using both manipulated and measured agency.

The results suggest that the perception that an individual posed a danger to society was a key predictor for why rights are withheld from mentally impaired defendants. This finding suggests that any ongoing harsh treatment of those with mental illness in the criminal justice system is not based on the desire to punish them *retrospectively* for their wrongdoing, as suggested by some scholars [32, 33], but rather, is the result of a *prospective* judgment of that individual’s capacity to be truly reintegrated into society as a moral agent. To the extent that these individuals are considered ‘unfit’ to return to society, they are stripped of their moral value as human beings. Crucially, the types of rights that mentally impaired defendants struggle to regain represent “invisible punishments” [43] that are not strictly demanded by sentencing policy (i.e., unrelated to one being found guilty or not guilty by reason of mental impairment) but rather, tools of moral and social exclusion. Our research thus goes beyond previous literature by showing a link between mental impairment and the determination of certain rights—one that cannot be entirely explained by generalized stigma towards those with mental illness (as demonstrated in Study 2) but, rather, is underlain by judgements of agency, and perceptions of continued threat.

Yet, re-establishing inhibitive agency can buffer the negative long-term impact of the defense. Our research suggests that that mentally ill offenders returning to the community need to show more than simply having “done their time” in order to recover their rights; they also need to demonstrate that they have regained inhibitive agency and are no longer a danger to others. We recognize that that this demonstration might be challenging. Forensic patients returning to their communities may be faced by community members who hold visceral and uninformed attitudes based on sensationalistic news reporting, myths, and stereotypes [32, 75–78]. Short of sharing individuals’ psychiatric records publicly, it is difficult to see how such persons would be able to convince their community that they have regained their agency. This points to the double burden on those who suffer from mental illness. That is, not only do they face multiple sources of disadvantage in their lives that can entangle them in the criminal justice system [79], but the burden of proof is higher for them to successfully reintegrate into society.

The current findings also provide new insights into the broader study of mind perception and morality. Previous research has downplayed the impact of agency on rights, and instead focused on the link between agency and responsibility [20, 25]. We found a nuanced perspective on the role of agency in moral rights. Generally speaking, when examining the most traditional definition of rights (moral standing), using the defense typically conferred an advantage to defendants. Moving beyond this basic negative rights definition, we also examined positive liberties, such as the right to marry, as well as the defendant’s rights to social services. Rights as citizens were those that appeared to take the biggest hit when using the defense, likely because these rights more naturally align with moral agency and criminal justice sanctions. And while patiency did play a role in conferring rights, indirect effects through inhibitive agency and dangerousness were consistently larger (Study 3), confirming the key role that agency plays in moral rights.

### Limitations and future directions

While we tested our model across two crime and two mental impairment contexts, we hesitate to generalize our finding to all cases. Nonetheless, we can tentatively suggest some conditions under which such a defense will be optimally effective. First, for a defense of mental impairment to be effective, the deficit in agency should be attributed to factors outside of the person’s control. If people are seen as responsible for their own mental illness they might still seem like they are agents, and mitigation of blame may not occur [22, 27, 80]. Therefore, conditions that are viewed as having a genetic or biological origin (and thus are outside the person’s control) should be more effective at reducing blame than conditions that are seen as a result of a person’s own actions [27, 81].

Next, for a defense of mental impairment to have the minimal negative downstream effects, the condition—and lack of agency implied by such—should be seen as either temporary or benign (e.g., a single isolated episode of psychosis). If a condition is perceived as chronic, then the revocation of rights should be more permanent (i.e., if they remain ill, then they may continue to be seen as unable to control their impulses and therefore dangerous). Indeed, we note in our supplemental analyses that there were some minor differences in the pattern of results between bipolar and brain injured defendants, with the outcomes slightly less favorable for brain injured defendants. It may be that brain injury was seen as less amenable to change, while the loss of agency brought about by bipolar may have been seen as flexible (e.g., through medication). More research would be required to unpack any possible differences in judgments across mental impairments.

On the other hand, it is also possible that lay people may overlook any such nuances. For instance, by the very act of amalgamating different types of mental illnesses under a single “mental impairment” label, it may be easy for observers to disregard the nature of defendants’ specific psychological impairment. More research is needed to test these possibilities.

In addition, future research could interrogate the conditions—or treatments—under which a mentally impaired defendant is seen to regain agency. In our recovered agency manipulation (Study 2), we did not specify how the mentally ill perpetrator was able to regain agency. Do different interventions equally restore perceived agency? For instance, if a person can manage their impulses, but only through the use of long-term medication, does this then restore perceived agency and rights? Or does society instead demand a deeper transformation of one’s agency—one that does not rely on medications or other external controls? In other words, where do people perceive agency (in particular, agency as relevant to the granting of rights) to be located—on the surface, or a deeper quality? Future research could identify the ways in which particular conditions of mental impairment and their corresponding treatments impact perceived agency, and thus, capacity for reintegration following the use of mental impairment defenses.

A final limitation relates to the sample used in this study Participants were exclusively United States residents; replication is required with culturally diverse samples. Even within Western democracies the United States is particularly punitive in its criminal justice policies [82]. It is possible that in countries with more rehabilitative penal attitudes, individuals will be more open to reintegrating defendants, including those who are mentally ill. Additionally, culture substantially shapes how people understand the nature and causes of mental illness [83, 84], which will likely shape perceptions of personal responsibility for mental illnesses (e.g., Ng [85] reports that mental illness in Japanese culture may be perceived as caused by a lack of willpower or self-control), as well as perceptions of dangerousness. Therefore, although we predict that our general pattern linking moral agency to responsibility, and dangerousness to reduced rights, would hold cross-culturally, the way that particular mental illnesses map onto agency, dangerousness, and rights, is likely to differ.

## Conclusion

The defense of mental impairment was designed with noble intentions in mind: to spare those who cannot be held responsible for their actions from the full weight of the criminal law. But raising mental impairment in a criminal case has implications well beyond what policy makers may have intended. Our research suggests that claiming a mental impairment may exonerate defendants of their crime, but by implying reduced agency, it may, in some cases, stymie the prospects of social reintegration in the longer term as it reduces perceived eligibility for moral rights. Additionally, our research challenges theoretical models in mind perception and rights, providing critical insights into the neglected link between agency and rights.

## Supporting information

S1 File (DOCX)
